# The Distribution and Influencing Factors of Hypolithic Microbial Communities in the Hexi Corridor

**DOI:** 10.3390/microorganisms11051212

**Published:** 2023-05-05

**Authors:** Yidan Zhao, Fasi Wu, Yang Liu, Minghui Wu, Shengjie Wang, Henry J. Sun, Guangxiu Liu, Yiyang Zhang, Xiaowen Cui, Wei Zhang, Tuo Chen, Gaosen Zhang

**Affiliations:** 1 State Key Laboratory of Cryospheric Sciences, Northwest Institute of Eco-Environment and Resources, Chinese Academy of Sciences, Lanzhou 730000, China; zhaoyd@lzb.ac.cn (Y.Z.);; 2 University of Chinese Academy of Sciences, No. 19A Yuquan Road, Beijing 100049, China; 3 Key Laboratory of Extreme Environmental Microbial Resources and Engineering, Lanzhou 730000, China; 4 National Research Center for Conservation of Ancient Wall Paintings and Earthen Sites, Department of Conservation Research, Dunhuang Academy, Dunhuang 736200, China; 5 School of Ecology and Environmental Sciences, Yunnan University, Kunming 650091, China; 6 Faculty of Geographical Science, Beijing Normal University, No. 19, Xinjiekouwai Street, Haidian District, Beijing 100875, China; 7 Desert Research Institute, Las Vegas, NV 89119, USA; 8 Key Laboratory of Desert and Desertification, Northwest Institute of Eco-Environment and Resources, Chinese Academy of Sciences, Lanzhou 730000, China

**Keywords:** hypolithic community, environmental factors, Gobi Desert, high-throughput sequencing, Hexi Corridor

## Abstract

The Hexi Corridor is an arid region in northwestern China, where hypoliths are widely distributed, resulting from large amounts of translucent stone pavements. In this region, the water and heat distributions are uneven, with a descent gradient from east to west, which can affect the area’s biological composition. The impact of environmental heterogeneity on the distribution of hypolithic microbial communities in this area is poorly understood, and this is an ideal location to investigate the factors that may influence the composition and structure of hypolithic microbial communities. An investigation of different sites with differences in precipitation between east and west revealed that the colonization rate decreased from 91.8% to 17.5% in the hypolithic community. Environmental heterogeneity influenced both the structure and function of the hypolithic community, especially total nitrogen (TN) and soil organic carbon (SOC). However, the effect on taxonomic composition was greater than that on ecological function. The dominant bacterial phyla in all sample sites were Cyanobacteria, Actinobacteria, Proteobacteria, and Deinococcus-Thermus, but the abundances varied significantly between the sampling sites. The eastern site had the highest relative abundance of Proteobacteria (18.43%) and Bacteroidetes (6.32%), while the western site had a higher relative abundance in the phyla Cyanobacteria (62%) and Firmicutes (1.45%); the middle site had a higher relative abundance of Chloroflexi (8.02%) and Gemmatimonadetes (1.87%). The dominant phylum in the fungal community is Ascomycota. Pearson correlation analysis showed that the soil’s physicochemical properties were also associated with changes in community diversity at the sample sites. These results have important implications for better understanding the community assembly and ecological adaptations of hypolithic microorganisms.

## 1. Introduction

Hypoliths are complex microbial assemblages, mostly found in biofilms; they are composed of autotrophs and heterotrophs that develop on the undersides of translucent stones in arid deserts [1,2,3,4,5]. A previous study found that almost every major desert pavement on Earth includes translucent stones, such as marble and quartz [6]. When higher plants and most other eukaryotes or prokaryotes cannot be supported, hypolithc microbial communities often show extremely high tolerance to drought and other environmental stressors due to the translucent stones that shield them from ultraviolet (UV) radiation [7,8]. Studies on hypoliths have shown that photosynthetic cyanobacteria are the most commonly dominant species in these communities [9,10,11]. They act as photoautotrophic microorganisms and primary producers, becoming the region’s main drivers [12,13]. They support hypolithic communities, occupying unique ecological niches and forming “islands in the sand” [4]. With the ongoing study of hypolithic microorganisms, there is increasing recognition of their importance in extreme desert systems.

Previous studies have shown that the environmental factors that may affect hypolithic microorganisms differ from region to region. These environmental factors typically include rainfall [14], liquid water effectiveness, fog [15,16], soil-chemistry-related variables [17], etc. For example, cyanobacterial abundance in the Atacama Desert decreased in hypolithic communities with decreasing annual precipitation [3]. In the Namib Desert, the community structures of hypolithic microorganisms changed with the fog–rainfall gradient [18]. Soil salinity is the main factor that influences differences in the hypolithic community’s structure and function between foggy and rainy areas in this region [17]. Site-based variations in the spatial patterns of cyanobacteria relates to precipitation in the Taklamakan Desert [10]. Studies in the desert of northwest China indicate that the diversity of hypolithic communities is largely influenced by the availability of liquid water, not by temperature or rainfall [19].

The Hexi Corridor is an arid region in northwestern China, where the Gobi Desert is widely spread. There is a clear change in the vegetation species from the city of Shandan to Yumen. Water and heat are unevenly distributed in this region. The monsoon climatic system primarily affects the eastern portion, whereas the westerlies primarily affect the central and western areas [20]. This results in significant spatial variation in annual precipitation. The aridity generally increases from the southeast to the northwest. In addition, influenced by precipitation, the highest Normalized Difference Vegetation Index (NDVI) values in the region are distributed in the southeast and are the lowest in the northwest [21]. Therefore, it is an ideal area to study the hypolithic microbial community. Previous studies have found strong seasonal variation in the hypolithic microbial community in this area. These are divided into cold and hot periods according to the changes in hypolithic microorganisms under different temperatures [22]. However, the previous study did not point out differences in geographical heterogeneity. Here, we selected sample sites with significant environmental variation along the Hexi Corridor from west to east and investigated the differences in the diversity, structure, and function of the hypolithic microbial communities by using high-throughput sequencing techniques. We identified two objectives: (i) to explore differences in hypolithic microbial colonization rates and community composition among samples, and (ii) to discuss the effects of environmental heterogeneity on the structure and function of the hypolithic community.

## 2. Materials and Methods

### 2.1. Study Area and Sampling

The Hexi Corridor region, which has a length of 900 km and a width of 100 km, with a total area of 215 × 10^5^ km^2^, is located in northwestern China. Owing to its temperate continental climate, the annual precipitation is 40–300 mm from west to east, and the mean annual air temperature is 6.2–9.0 °C [21,23].

**Figure 1:**
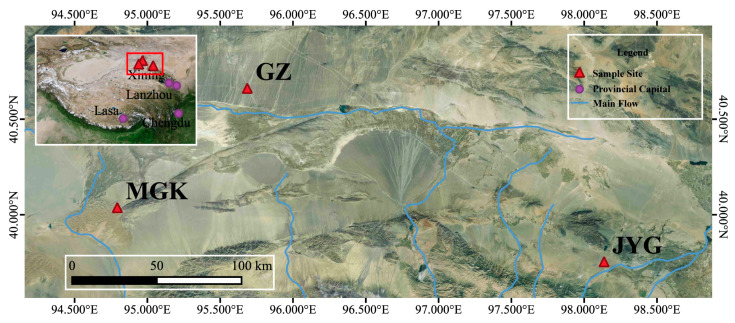
Spatial distribution of the sampling sites in the Hexi Corridor, China.

Samples were collected at 3 locations over a distance of approximately 550 km (39.45–40.20° N, 94.47–98.80° E) in June 2021; these locations were the Jiayuguan Gobi Desert (JYG), the North of Guazhou Gobi Desert (GZ) and the Mogao Grottoes desert (MGK) (Figure 1). These areas were covered with large amounts of gravel rocks (Figure S1a), including translucent stones that usually have green or yellowish-green patches and rings of pigment where hypolithic microbial communities grow (Figure S1b). Hypolithic microbial samples were sealed in sterile sampling bags (EFR-5590E, LABPLAS, TWIRL’EM^®^, Montreal, QC, Canada). Meanwhile, we collected soil from beneath a number of stones (approximately 1 cm thick) at each sampling site until a sterile 50 mL sampling tube was filled, with 3 replicates per sample site. All samples were stored in a cold, dry, and dark sampling box with an ice pack.

It is worth noting that cyanobacteria have a shared evolutionary history with chloroplasts. We minimized chloroplast contamination during sample collection. At our sampling sites of MGK and GZ, there were nearly no plants, and the hypoliths colonizing stones at the JYG site were paved in the interspace of the plants (Figure S1). During the sample collection, the stones with colonized hypoliths were tightly adhered to the ground and had no space for chloroplast contamination from plant litter.

### 2.2. DNA Extraction and Gene Sequencing

DNA extraction for all samples was processed immediately upon return to the laboratory. Under sterile conditions, the hypolithic microbial communities attached to the rock surface were rinsed with a sterile cotton swab in a 2 mL sterile centrifuge tube, and 0.8 g of each collected soil sample was weighed.

Microbial total DNA was extracted using a FastDNA^®^ Spin Kit for soil (MP Biomedicals, New York, NY, USA) according to the manufacturer’s instructions. In the bacterial 16S rRNA V3-V4 region, amplification was performed using forward primer 338F and reverse primer 806R [24]. The fungal ITS region was amplified by the forwarding primer ITS1F and the reverse primer ITS2R [25]. The PCR reaction cycling conditions were as follows: initial denaturation at 95 °C for 3 min, followed by 30 cycles of denaturation at 95 °C for 30 s, annealing at 72 °C for 45 s, and final elongation at 72 °C for 10 min.

The amplicons were sequenced using the Illumina MiSeq platform (PE300, Illumina, San Diego, CA, USA). The raw sequences underwent quality control using Trimmomatic and were merged by FLASH [26] according to the following criteria: (i) the 300 bp reads were truncated at any site that received an average quality score of <20 over a 50 bp sliding window, and the truncated reads shorter than 50 bp were discarded, as were reads containing ambiguous characters. (ii) Only overlapping sequences longer than 10 bp were assembled according to their overlapped sequence. The maximum mismatch ratio of the overlap region is 0.2. Reads that could not be assembled were discarded. (iii) Sequences were distinguished according to the barcode and primers, and the sequence direction was adjusted for exact barcode matching, with 2 nucleotide mismatches in primer matching. Operational taxonomic units (OTUs) and chimeras that were grouped based on 97% similarity were removed using Uparse [27]. The OTU table was manually filtered, i.e., chloroplast sequences in all samples were removed. To minimize the effects of the sequencing depth on alpha and beta diversity measures, the number of 16S rRNA gene sequences from each sample was rarefied to 20,000. The RDP classifier [28] (http://rdp.cme.msu.edu/, accessed on 24 March 2022, version 2.11) was used to compare the Silva [29] 16S rRNA gene database (v.138) and the Unite (8.0) [30] database for OTU species taxonomic annotation with a confidence threshold of 70%. The raw sequencing reads were deposited into the Genome Sequence Archive (GSA) (Accession Number: CRA008403).

### 2.3. Measurements of Colonization, Soil Physicochemical Characteristic Analyses, and Environmental Data Collection

In order to determine the colonization rate, a 1 m × 10 m quadrat was selected to count whether the microbial community colonized the undersides of the translucent stones in the quadrat. The number of translucent stones within each sample square was at least 100, with 10 replicates per sample site [3].

To determine the soil’s physicochemical characteristics, the soil was naturally air-dried, ground, and graded. All physiochemical measurements were repeated three times for each soil sample. Soil organic carbon (SOC) was determined using the potassium dichromate titration method. The soil total phosphorous (TP) content was determined using the molybdenum antimony colorimetric method [31]. Total nitrogen (TN) contents were determined using the Kjeldahl method [32]. Soil pH was measured using a pH meter (PT-10, Sartorius, Göttingen, Germany) with the ratio of fresh soil: water = 1:2.5 (*w*/*v*). Electrical conductivity (EC) was measured using a conductivity meter (DDSJ-308A, Leici, Shanghai, China). Soil K^+^, Na^+^, Ca^2+^, and Mg^2+^ contents were determined with the ratio of fresh soil: water = 1:5 (*w*/*v*) using an Atomic Absorption Spectrometry analyzer (AAS) (Thermo Fisher Scientific, Waltham, MA, USA). HCO^3−^ was determined by chemical titration with 0.02 mol/L sulfuric acid. Cl^−^ was determined using the chemical titration method with 0.04 mol/L silver nitrate. SO_4_^2−^ was determined by EDTA indirect titration [33].

Environmental data, including the 2010 to 2019 mean average temperature, precipitation, and vegetation index data, were collected (including data for each month). Mean annual precipitation and mean annual temperature data were obtained from the University of East Anglia Climate Research Centre (https://www.uea.ac.uk, accessed on 7 July 2022) and vegetation index data (resolution 0.5) were obtained from the National Environmental Information Center (https://www.ncei.noaa.gov/, accessed on 7 July 2022).

### 2.4. Keystone Species Analysis and Function Analyses

For the analysis of key species at different sites, we describe the distribution of bacterial and fungal species’ levels based on their respective network roles (SparCC network) [34]. LEfSe analysis (linear discriminant analysis effect size) [35] (http://huttenhower.sph.harvard.edu/LEfSe, accessed on 26 January 2023) (LDA > 3.5, *p* < 0.05) was used to identify bacterial and fungal species with significantly different abundance between groups, from phylum- to genus-level taxa that differed significantly in abundance from the phylum to the genus level between different sites.

A co-occurrence network was constructed using the “WGCNA” R package based on the Spearman correlation matrix [36]. To reduce the number of rare OTUs in the data set, we removed OTUs with zero abundance in more than 80% of the samples from the bacterial and fungal sequences. A SparCC network was constructed using the R language, ‘SpiecEasi’ package and sparcc function (https://rdocumentation.org/packages/SpiecEasi/versions/1.0.7, accessed on 1 November 2022). Based on the SparCC network, to define the node types for network hubs (z-score > 2.5; c-score > 0.6), module hubs (z-score > 2.5; c-score > 0.6), connectors (z-score 2.5; c-score > 0.6), and peripherals (z-score 2.5; c-score 0.6) according to their functions in the network structure, we removed the edges in the network with *p* > 0.05, and |r| < 0.6. The co-occurrence network’s crucial nodes were determined using the values of within-module connectivity (Zi) and among-module connectivity (Pi) [37].

Two software tools that forecast the function of microbial communities were used to carry out the functional analyses. The OTU abundance tables of the three-site samples were normalized using PICRUSt2 (the Phylogenetic Investigation of Communities by the Reconstruction of Unobserved States) [38], i.e., to remove the effect of the number of copies of the 16S marker gene in the species genome; the KEGG Ortholog (KO) information corresponding to the OTU was then obtained from the Greengene ID corresponding to each OTU. FAPROTAX (the Functional Annotation of Prokaryotic Taxa) was used for the functional annotation prediction of the biogeochemical cycling capacity for the environmental DNA samples, especially sulfur, nitrogen, hydrogen, and carbon cycles.

### 2.5. Statistical Analyses

Sampling sites were mapped using ArcGIS (version 3.22.5). The variance of environmental factors and microbial alpha diversity were determined using SPSS 26 (IBM SPSSInc Software, Armonk, NY, USA), and histogram figures were plotted using Origin2019 (Origin Lab 2019, Northampton, MA, USA). When *p* < 0.05, Tukey’s test was used to assess whether there was a significant difference between treatments. Statistical analysis of the OTUs was conducted using Usearch 7 [39]. R was used to examine microbial alpha diversity (Veen Diagram package). Utilizing Mothur [40] 1.30.2, OTU Venn analyses of several biomes were conducted. The beta diversity distances were calculated using QIIME 1.9.1, and pairwise categorical distance matrices between bacterial communities were analyzed using the Bray–Curtis method. Principal coordinate analysis (PCoA) of the community structure and functional predictions of microbial communities at the three sites were conducted using the ‘ape’ and ‘vegan’ packages of RStudio software; figures were generated by the ‘ggplot2′ package, and between-group difference analysis was performed using PERMANOVA. The sample sites for detrended correspondence analysis (DCA) and redundancy analysis (RDA) were calculated using the R language ‘vegan’ package OTU, and CCA plots and RDA plots were selected to represent the environmental correlation within the sample.

## 3. Results

### 3.1. Environmental Factors and Colonization Rates

**Figure 2:**
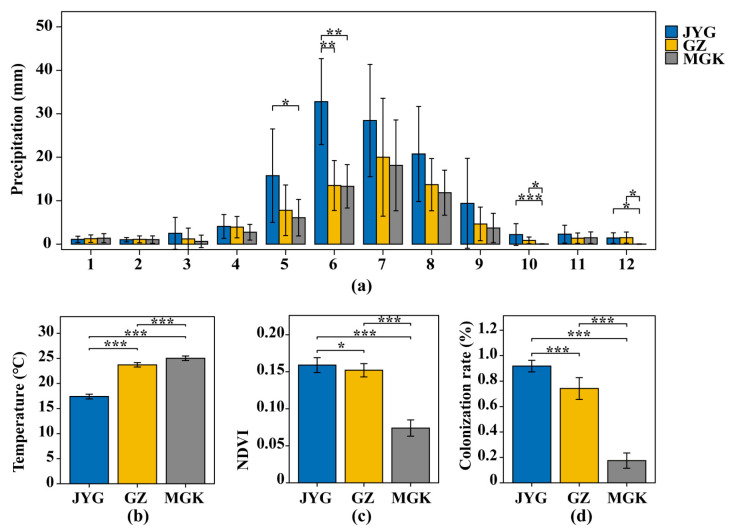
Comparison of environmental factors and the colonization rate of hypolithic communities among the three sample sites. (**a**) Monthly precipitation differences among the three sample sites. The horizontal coordinate indicates January to December; (**b**) mean annual air temperature in summer for the last ten years at the three sample sites; (**c**) mean annual normalized difference vegetation index of the three sample sites; (**d**) a comparison of colonization rate in June at the three sample sites. The symbol * refers to *p* < 0.05; ** refers to *p* < 0.01; *** refers to *p* < 0.001.

According to the Normalized Difference Vegetation Index (NDVI) and meteorological data, the mean annual precipitation (MAP) rates of JYG, GZ, and MGK were 121.76 mm, 70.86 mm, and 60.45 mm, respectively. More than 78% of the annual precipitation in the sample sites is concentrated from May to September (Figure 2a). The rest of the months have low precipitation, with the average monthly precipitation not exceeding 5 mm. The vegetation cover at the three sampling sites gradually decreased from JYG to MGK (Figure 2c), and the air temperature increased (Figure 2b). The mean annual air temperature in summer (June to August) differed significantly between the sites, with MGK (25.03 °C) having the highest value and JYG (17.4 °C) the lowest. The NDVI of MGK was 0.74, which was lower than half of the average of the other two sites. The colonization rates of JYG, GZ, and MGK were 91.8%, 74.2%, and 17.5%, respectively (Figure 2d). The correlation analysis between the precipitation and colonization rates in different months is shown in Table S1.

### 3.2. The Physico-Chemical Properties of the Soil Samples

**Figure 3:**
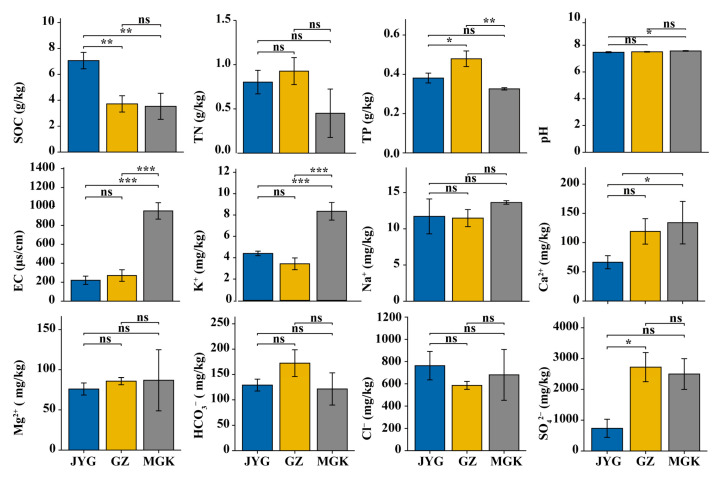
Soil physiochemical properties of the three sampling sites. SOC, soil organic carbon; TN, soil total nitrogen; TP, soil total phosphorous; EC, electrical conductivity. Values are the mean ± SD. The symbol ns refers to *p* ≥ 0.05; * refers to *p* < 0.05; ** refers to *p* < 0.01; *** refers to *p* < 0.001.

The differences in the soil properties of the three sample sites related to the soil organic carbon (SOC), electrical conductivity (EC), soil total phosphorous (TP), and K^+^, Ca^2+^, and SO_4_^2−^ concentrations. The pH of the soil at all the sampling sites was close to neutral, with values around 7.5. The EC varied between 270.33 to 999.50 μs/cm; among them, the value for site MGK was significantly higher than those for the other two sites. For SOC, site JYG was higher than sites GZ and MGK, with values of 7.06 g/kg, 3.72 g/kg, and 3.53 g/kg, respectively. The TP of GZ was the highest, with values of 0.48 g/kg. For K^+^ and Ca^2+^, site MGK has the highest value, and the variation ranges of the 3 sites were 3.43 to 8.35 mg/kg and 66.33 to 134.08 mg/kg, respectively. Site JYG had the lowest value for SO_4_^2−^, 736 mg/kg. There were no significant differences in total nitrogen (TN) or Na^+^, Mg^2+^, HCO_3_^−^, and Cl^−^ concentrations between the three sites (Figure 3).

### 3.3. Hypolithic Microbial Community

After assembly and quality filtering, a total of 8,609,701 high-quality sequences were obtained from the whole sequencing dataset; these were clustered into 5977 operational taxonomic units (OTUs), affiliated to 29 phyla, 80 classes, 194 orders, 309 families, 582 genera, and 1095 species.

**Figure 4:**
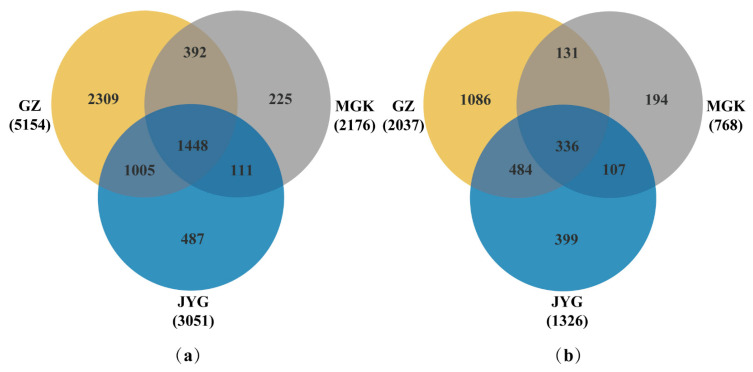
Venn diagrams of the bacteria (**a**) and fungi (**b**) at the three sites, showing the common and unique OTUs.

A Venn diagram demonstrates the similarities and differences in OTUs between the three sites (Figure 4). In the bacterial communities (Figure 4a), there were 487, 2309, and 225 site-specific OTUs at the JYG site, the GZ site, and the MGK site, respectively. Only 1448 of the 10,381 OTUs were shared across all 3 plots, accounting for 13.95% of the total OTUs in the fungal community (Figure 4b); the numbers of site-specific OTUs were 399, 1086, and 194 for the different sites, respectively. Only 336 out of 4131 OTUs were shared across all three sites (8.13% of the total OTUs).

In the bacterial communities, the predominant phyla of the three sites were Cyanobacteria, Actinobacteria, Proteobacteria, and Deinococcus-Thermus, with relative abundances of 48.06%, 14.88%, 18.37%, and 5.8% in the JYG site, 50.84%, 16.6%, 9.75%, and 6.1% in GZ site, and 61.96%, 16.49%, 8.48%, and 4.66% in MGK site, respectively. They were followed by members of Chloroflexi (3.16%, 3.12%, and 8.04%), Bacteroidetes (6.31%, 1.84%, and 5.19%), and Gemmatimonadetes (1.1%, 1.62%, and 1.88%) (Figure S2a). JYG had the highest relative abundance of Proteobacteria, Bacteroidetes, and Actinobacteria. GZ had a higher relative abundance of Chloroflexi and Gemmatimonadetes. MGK had a higher relative abundance of the phyla Cyanobacteria and Firmicutes.

In the fungal communities, the dominant phyla at the three sites were Ascomycota, at rates of 89.41%, 91.44%, and 81.76%. Basidiomycota had rates of 2.13%, 3.4%, and 6.34%. The relative abundance of Chytridiomycota in GZ was 2.24%, and it was a rare phylum at the other two sites (Figure S2b). The relative abundance of bacteria and fungi on the genus level showed differences in terms of their relative abundance at the three sites (Figure S2c,d).

### 3.4. Alpha Diversity Index of the Hypolithic Microbial Community

**Figure 5:**
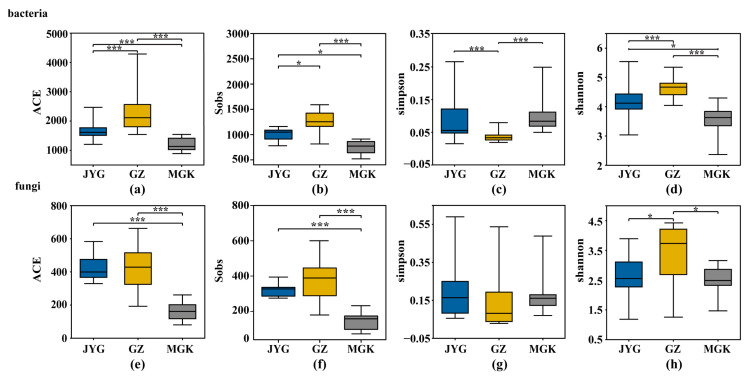
Bacterial and fungal α-diversity indices for the three sample sites. The richness of bacteria and fungi was expressed by the AEC (**a**,**e**) and Sobs (**b**,**f**) indices, respectively. The diversity of bacteria and fungi was expressed by the Simpson (**c**,**g**) and Shannon (**d**,**h**) indices, respectively. The symbol * refers to *p* < 0.05; *** refers to *p* < 0.001.

In the bacterial communities, ACE (an estimated species richness based on the Abundance-based Coverage Estimator) demonstrated the same trend as the Sobs (the observed species richness) results (Figure 5a,b). Species richness was significantly different across the three sites (Figure 5a), with GZ having the highest level (2191.52), followed by JYG (1632.35), while MGK had the lowest (1180.77). The Shannon diversity index (which takes into account both the species’ richness and their relative abundance) of the GZ site was significantly higher than that of the other two sites; it was followed by JYG, while the MGK site had the lowest value (*p* < 0.001) (Figure 5d). In the fungal communities, the differences in species richness between the GZ and JYG sites were small, and the MGK site was significantly lower, with an ACE value of 161.31. The actual observed OUT number also showed a consistent level (Figure 5e,f). Combining the Shannon and Simpson indices (a means of measuring the diversity of species in a community) showed that the diversity of the GZ site was higher at the fungal level (Figure 5h,g). In summary, the GZ sample site had the highest abundance and diversity of hypolithic bacterial and fungal communities, while the MGK site had the lowest. The richness of the community at the GZ sample site was not related to the number of samples (Figure S3).

According to Pearson correlation, the pH, EC, and K^+^, Na^+^, and Cl^−^ concentrations were significantly negatively correlated with fungal and bacterial ACE values and the Shannon diversity. TN, TP, and HCO_3_^−^ were significantly positively correlated with fungal and bacterial ACE and Shannon diversity. Ca^2+^ and Mg^2+^ were only negatively correlated with fungal abundance. SOC and MAP were only positively correlated with fungal abundance (Table 1).

### 3.5. Beta Diversity of the Hypolithic Microbial Community and Functional Predictions

To understand the impact of geographic location on microbial communities, we performed principal coordinate analysis (PCoA) (Figure S4). It was found that the structure of the hypolithic microbial community at the three sites on the PC1 axis could explain the geographic differences in bacterial communities (Figure S4a,b). The community structure of bacterial and fungal PERMANOVA results showed R^2^ = 0.4 and 0.3, respectively. However, from the perspective of community function, the variation in bacterial groups at the three sites was less than the community structure of bacteria and fungi (Figure S4c,d), with PERMANOVA results of R^2^ = 0.28 and 0.29, respectively.

**Figure 6:**
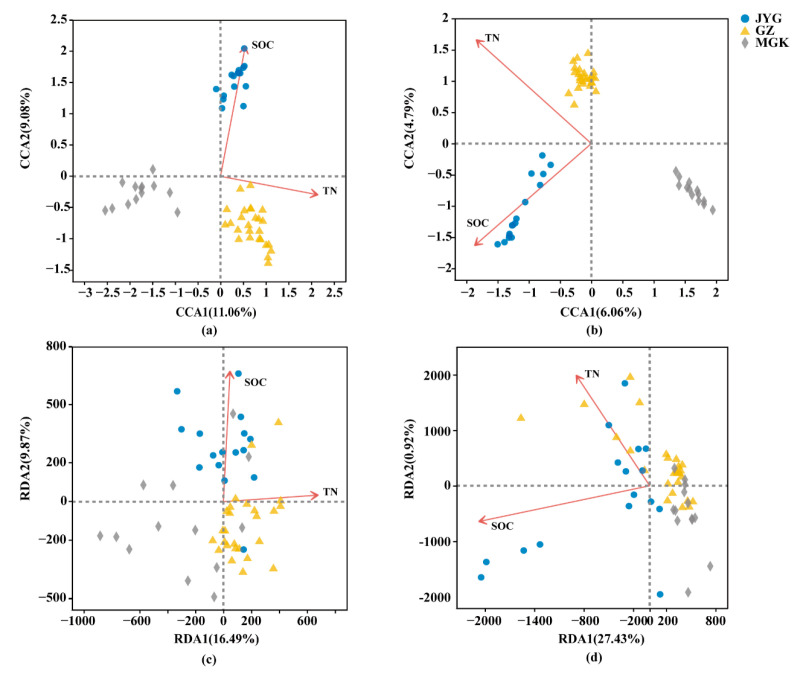
Plots based on the canonical correspondence analysis (CCA) and redundancy analysis (RDA) of pyrophosphate; sequenced 16S rRNA and ITS genes were obtained from the three sample sites. Physiochemical data included soil anions and cations, soil pH, electrical conductivity, air temperature, the NVDI index, mean monthly precipitation, and mean annual precipitation. (**a**,**b**) The 16s/ITS rRNA-based calculations; (**c**,**d**) KEGG table calculations based on functional predictions. (**a**,**c**) For the bacterial community and (**b**,**d**) for the fungal community. The percentages explained by each axis are represented by the values of axes 1 and 2.

Redundancy analysis (RDA) demonstrated that the microbial community structure and the results of functional predictions were determined by major environmental characteristics, including soil organic carbon (SOC) and total nitrogen (TN) (Figure 6). Other environmental factors have no obvious correlation with the microbial community’s structure.

Functional predictions based on PICRUSt2 identified differences in the bacterial and fungal functions between the three sites, mainly involving lipid metabolism, energy metabolism (mainly photosynthesis), and the replication and repair of genetic information (Figure S5a,c).

In the fungal community, the abundance of some enzymes increased from JYG to MGK, such as Adenosine triphosphatase, Glucan 1,4 alpha-glucosidase, DNA-directed RNA polymerase, H(+)-transporting two-sector ATPase, Histone acetyltransferase, 1 alkyl 2-acetylglycerophosphocholine esterase, and DNA ligase (ATP). The abundance of NADPH (quinone reductase), Glutathione transferase, NAD(+) ADP-ribosyltransferase, flavin-containing monooxygenase, Lysophospholipase, Histone-lysine N-methyltransferase, and Acylglycerone-phosphate reductase decreased in terms of abundance (Figure S5b). There were significant differences between xylanolysis, cellulolysis, and chemoheterotrophy in the hypolithic communities (Figure S5d).

### 3.6. Keystone Species Analysis

**Figure 7:**
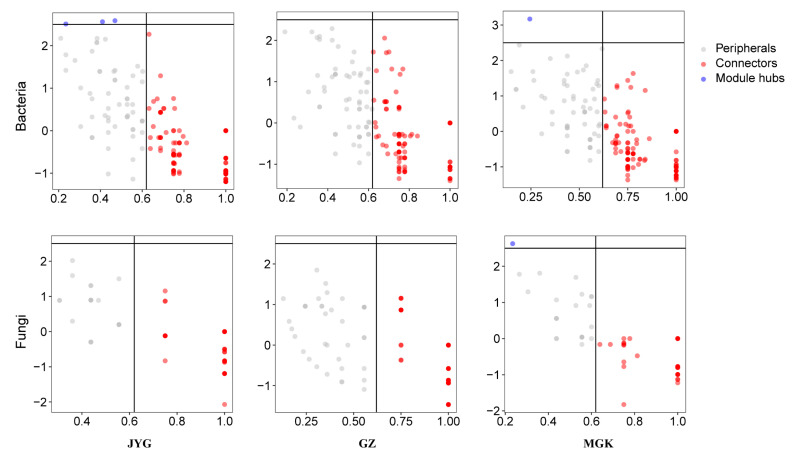
Actors and regions in the z-P parameter space; see Methods for definitions.

In the bacterial communities, JYG had 1.8% in module hubs and the connectors had 63.29%; MGK had 0.63% in module hubs and no module hubs were found in GZ (Figure 7). LEfSe analysis showed that the JYG site had higher concentrations of the order Rhizobiales, the families Chroococcidiopsaceae, Coleofasciculaceae, Beijerinckiaceae, Acetobacteraceae, Caulobacteraceae, and Blastocatellaceae, and the genera Psychroglaciecola and Microvirga. The GZ site had an abundance of the orders Cyanobacteriales, Chroococcidiopsaceae, and Rubrobacteriaceae, the orders Bacillales, Frankiales, and Micrococcales, and the families Micrococcaceae and Phormidiaceae. The genera of Arthrobacter and Kocuria were more abundant at the MGK site (Figure S6a).

In the fungal communities, only MGK exhibited module hubs, with a value of 0.84% (Figure 7). LEfSe analysis showed that the JYG site had higher concentrations of the families Pleosporaceae, Sporormiaceae, and Ascobolaceae, and the genera Preussia, Alternaria, Ulocladium Ascobolus, and Iodophanus. The GZ site had an abundance of the families Didymellaceae, Rhizophlyctidaceae, Didymosphaeriaceae, Phelloriniaceae, Periconiaceae, and Plectosphaerellaceae (Figure S6b).

## 4. Discussion

Precipitation plays a key role in the colonization of hypolithic communities. As annual precipitation decreased, the results of our field survey in June showed that colonization rates in the hypolithic community were also decreasing, which is consistent with results previously obtained for September in the same area [22]. One possible reason for this is that such minor changes within the community may not be seen with the naked eye; the outcomes of such observations might remain consistent throughout the year. In addition, different cyanobacteria and other biofilm-forming bacteria on rocks may be connected to biofilms that adhere to rocks [41]. Even if certain bacterial communities die due to harsh conditions, these biofilms do not disappear in the short term.

Correlation analysis showed that monthly precipitation from May to July and in October and December was significantly and positively correlated with the hypolithic colonization rate (Table S1). This phenomenon seems to be closely related to the seasonal growth of the hypolithic community [22]. The precipitation events in all three sample sites were concentrated from May to September (Figure 2). Although there is little direct evidence of seasonal variation in hypolithic communities, several studies have also indicated the importance of seasonal precipitation on community growth to certain extent. As extracellular polymeric substrates (EPS) inflate with water and the cells are ejected and transported to different regions on the rock, lithic cyanobacterial colonies on specific rocks spread during wet seasons [10,42,43]. This partially explains the association between the season with the highest correlation of precipitation and the rate of hypolithic colonization. The growing seasons for arid hypolithic communities are very limited. Some hypolithic communities are capable of dormancy under dry conditions while they wait for wetting events to occur [44]. In cold, dry environments, such as polar deserts, exposed rocks may promote moisture concentration by trapping wind-blown ice crystals and snow [5]. In addition, the availability of liquid water is a key factor that cannot be ignored in hypolithic communities [3]. This may imply that winters with low precipitation may be active periods for the growth of some photosynthetic autotrophs (e.g., cyanobacteria) that can survive under cold conditions, rather than heterotrophic microorganisms.

Soil physicochemical properties have a significant impact on both the composition structure and function of hypolithic communities. In this study, we demonstrated that the soil’s physicochemical properties at three sample sites in the Hexi Corridor had a strong influence on the abundance and diversity of bacteria and fungi. The MGK sample site had the highest EC and a high salinity concentration. The high soil conductivity indicates a chronic lack of liquid water in MGK. Thus, the negative correlation between conductivity and abundance may reflect that this site is under prolonged drought conditions, which are insufficient to support the growth of some heterogeneous bacteria with high nutrient requirements. The role of pH in influencing soil bacteria is not surprising, with most soil pH values ranging from 7 to 10; soil pH has a strong influence on community composition. The closer the soil to neutrality, the higher the diversity of the soil bacteria; this is due to the direct physiological constraints of pH on soil microorganisms, many of which have an intracellular pH close to neutral and cannot survive once it falls below a certain range. Changes in the structure and function of hypolithic microbial communities are influenced by the physicochemical properties of the soil (Figure 6).

Spatial patterns, such as increased geographic distance, have been reported to lead to the diversity of hypolithic communities [45]. This may be related to topography, soil or rock properties, water effectiveness, etc. [10]. Our study shows that SOC and TN have effects on community structure and, to a lesser extent, function. The presence of different taxonomic species with the same function may have produced such a result [46]. Changes in stoichiometric imbalances can also lead to changes in the microbial community’s composition due to differences in the resource requirements, growth, and competition and adaptation strategies of different microbial taxa [47]. SOC had a greater effect on bacteria and fungi in the JYG community, and TN had a greater effect on bacteria and fungi in the GZ community. Some heterotrophic bacteria with high nutrient requirements usually dominate in soil habitats containing high-quality (low C:N and C:P ratios) substrates [47]. In this study, the GZ sample site’s soil had the lowest C:N and C:P ratios.

Photoautotrophic communities play a crucial role as primary producers in such a harsh environment [10,12]. The relative abundance of the Cyanobacteria phylum increased at the relatively arid MGK sampling site, a phenomenon unlike that observed in the extremely arid Atacama Desert [3]. These studies show that cyanobacteria can grow in areas with high salt ion concentrations, that many cyanobacteria are moderately salt tolerant, and that nitrogen-fixing cyanobacteria have been used to ameliorate coastal soil salinity [48]. Certain taxonomic groups such as *Rubrobacter* and *Nostoc*, with tolerance to oxidative stress, have the ability to colonize deteriorated rocks; they are also able to increase their uptake of K^+^ at lower concentrations, which helps them to survive in such harsh conditions, since the soil K^+^ concentration depends to a large extent on the soil nutrient status, mineral type, etc. Some bacteria of the genus *Rubrobacter* are also able to tolerate high temperatures, and studies have isolated many thermophilic species of this class, all of which have optimal growth temperatures above 50–60 °C [49]. Members of the genus *Chroococcidiopsis*, such as *Chroococcidiopsis* (CC1) and *Chroococcidiopsis* (SAG_2023), differed significantly between the three sample sites. The former is classified as clade I, belonging to the cold desert taxon, and the latter is classified as clade II, belonging to the hot desert taxon [19]. Interestingly, *Chroococcidiopsis_CC1* is only present at MGK as a connector. *Chroococcidiopsis_SAG_2023* is only present at GZ as a key connector species. There are also some members of the cyanobacterial phylum Leptolyngbya, genus Crinalium, with significantly high abundance in the MGK site. In addition, some rare members of Actinomycetaceae, such as the small clover family and the mycorrhizae, have become keystone species at MGK.

Some non-cyanobacterial phototrophic taxa, Chloroflexi, were found at all sample sites. We observed the highest abundance of Chloroflexi in the GZ site. It has been reported to be more effective than cyanobacteria in fixing nitrogen and is able to fix carbon [50]; it therefore plays a vital role in the freeze–thaw and dehydration tolerance of the community and dominates the hyper-arid Atacama Desert [13]. This may be linked to the green tint present on the sandstone surfaces, which could be related to the extensive colonization area of this sample of hypolithic bacteria on individual rocks [51]. Another clade with a high abundance in the GZ site is the Gemmatimonadetes member, which has been shown to thrive in carbon- and nitrogen-deficient conditions and often occurs in the root nodules of plants [52,53]. A detail that is indicative of this result is that, with the spatial variation of the precipitation gradient, the vegetation at the three points became increasingly sparse, with not even a single bush at MGK. In addition, *Microcoleus*, *Oscillatoria*, and *Scytonema* were found to be widespread in the three sample sites, with their strong drought-tolerance mechanisms involving motility, the production of extracellular polysaccharides, etc. [54].

In terms of microbial functions, photosynthesis, metabolism, and some defense and repair functions were increased at the MGK site, while heterotrophic and some defense and repair functions at the level of secondary metabolites were decreased. The highest abundance of cyanobacteria found in the MGK sample site may be associated with an increase in photosynthesis. Cyanobacteria may invest more energy in DNA repairs in the harsh environments of the driest place [55].

The fungal communities showed lower geographical fluctuations, lower abundance, and less variety than the bacterial communities, suggesting that fungi may respond to environmental changes by changing their rare taxonomic units. Precipitation and SOC only influenced the abundance of fungi. The study showed that, significantly, all three sample sites had the highest relative abundance of Ascomycota, the largest fungal phylum, with between a third and a half of the ascomycete species being associated with lichen forms of algae or cyanobacteria [56]. Cyanobacteria breakdown can transmit nutrients to the surrounding environment, but other fungi (e.g., the Stenotrophomonas phylum) can take up nutrients by feeding on decaying material; they therefore play an essential part in the biogeochemical carbon and nutrient cycles [57].

The composition of bacterial and fungal communities is not only influenced by broad environmental patterns but also by microhabitat responses, as suggested by previous reports [57]. This requires further study in terms of hypolithic microorganisms.

## 5. Conclusions

We investigated the differences in the composition and structure of hypolithic bacterial and fungal communities at three sampling sites at different spatial levels in the Hexi Corridor. It was found that the soil’s physicochemical properties at different sites influenced the richness and diversity of the hypolithic communities. Among them, both TN and SOC significantly influenced the structure and, to a lesser extent, function of the hypolithic community at the three sample sites. Cyanobacteria dominated the hypolithic community at the three sample sites, and the fungi mainly comprised ascomycetes. Our study strengthens the current understanding of the patterns of the fungal and bacterial communities in the hypolithic communities of the Hexi Corridor. This work is expected to facilitate the further analysis of the dynamics of the lithic communities during different seasons. Hypolithic habitats are characterized by different ecological conditions to those that occur at the soil’s surface layer, which may have important implications for the characteristics of hypolithic microorganisms.

## Figures and Tables

**Table 1:** Pearson correlation coefficients of fungal and bacterial diversity with environmental factors.

Environment	Bacteria		Fungi	
	ACE	Shannon	ACE	Shannon
pH	−0.380 **	−0.422 **	−0.691 **	−0.152
EC	−0.535 **	−0.564 **	−0.731 **	−0.279 *
SOC	−0.145	−0.09	0.315 *	−0.217
TN	0.658 **	0.669 **	0.694 **	0.401 **
TP	0.691 **	0.679 **	0.530 **	0.476 **
MAP	−0.098	−0.043	0.359 **	−0.188
K^+^	−0.637 **	−0.653 **	−0.711 **	−0.377 **
Na^+^	−0.608 **	−0.628 **	−0.724 **	−0.347 **
Ca^2+^	0.025	−0.029	−0.424 **	0.14
Mg^2+^	0.112	0.057	−0.346 **	0.197
Cl^−^	−0.486 **	−0.442 **	−0.087	−0.414 **
HCO_3_^−^	0.669 **	0.648 **	0.438 **	0.482 **

Notes: EC, electrical conductivity; SOC, soil organic carbon; TN, soil total nitrogen; TP, soil total phosphorus; MAP: mean annual precipitation; Codes: *p* < 0.05 (*), *p* < 0.01 (**), *p* > 0.05 (blank).

## Data Availability

All the resources can be accessed at https://ngdc.cncb.ac.cn, accessed on 25 November 2022, CRA008403.

## Supplementary Materials

The following supporting information can be downloaded at: https://www.mdpi.com/article/10.3390/microorganisms11051212/s1, Figure S1: Typical landscapes of the Gobi Desert (a). Hypolithic microorganisms colonized close to the bottom of the translucent rock (b); Figure S2: The relative abundance of bacterial (a) and fungal (b) phyla present in the hypolithic community; Figure S3: Cumulative curve for bacteria and fungi at three sites. The horizontal coordinate indicates the number of samples; Figure S4: Principal coordinates analysis (PCoA) based on Bray–Curtis distances shows the bacterial community (a), fungal community (b), bacterial function (c), and fungal function (d) among the three sample sites; Figure S5: Analysis of the differences in the function of bacteria (a) and fungi (b) at three sites. The graph shows the results for *p* < 0.001 and the top 30 in terms of abundance. Prediction of ecologically relevant functions for bacteria (c) and fungi (d) based on the FAPROTAX. * Refers to *p* < 0.05; ** refers to *p* < 0.01; Figure S6: LDA Effect Size (LEfSe) algorithm was used on phylum to genus level to bacterial (a) and fungal (b) OTU tables. Only taxa meeting an LDA significance threshold >3.5 are shown; Table S1: Spearman correlation coefficients between precipitation and the colonization rate in different months.
