# Supplementary material for: The Distribution and Influencing Factors of Hypolithic Microbial Communities in the Hexi Corridor
Source: Microorganisms. 2023 May 5;11(5):1212. doi: 10.3390/microorganisms11051212 (PMC10223714; doi:10.3390/microorganisms11051212)
Supplement: Supplementary file 1 [file microorganisms-11-01212-s001.zip › microorganisms-2336173-supplementary.pdf]

microorganisms

MDPI

Table S1. Spearman correlation coefficients between precipitation and colonization rate in different months.

Precipitation (mm)	1	2	3	4	5	6	7	8	9	10	11	12
colonization rate (%)	0.094	0.135	0.319	0.318	0.396*	0.613**	0.407*	0.291	0.171	0.770**	0.129	0.527**

Notes: 1 to 12 means January to December. Codes: $p < 0.05$ (*), $p < 0.01$ (**).

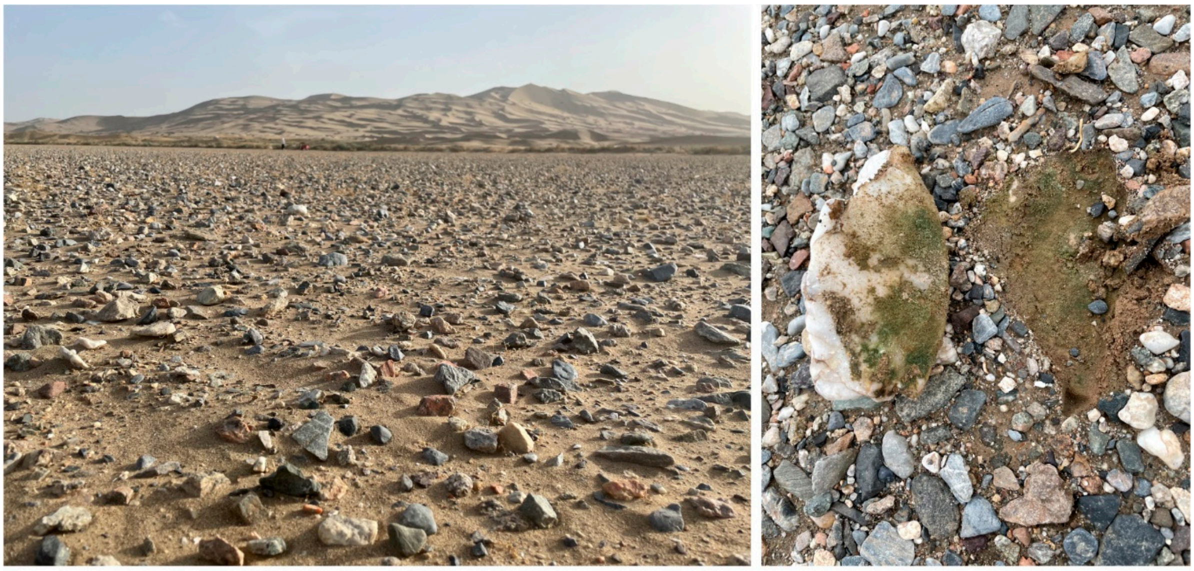

(a) (b)

Figure S1. Typical landscapes of the Gobi Desert (**a**). Hypolithic microorganisms colonized close to the bottom of the translucent rock (**b**).

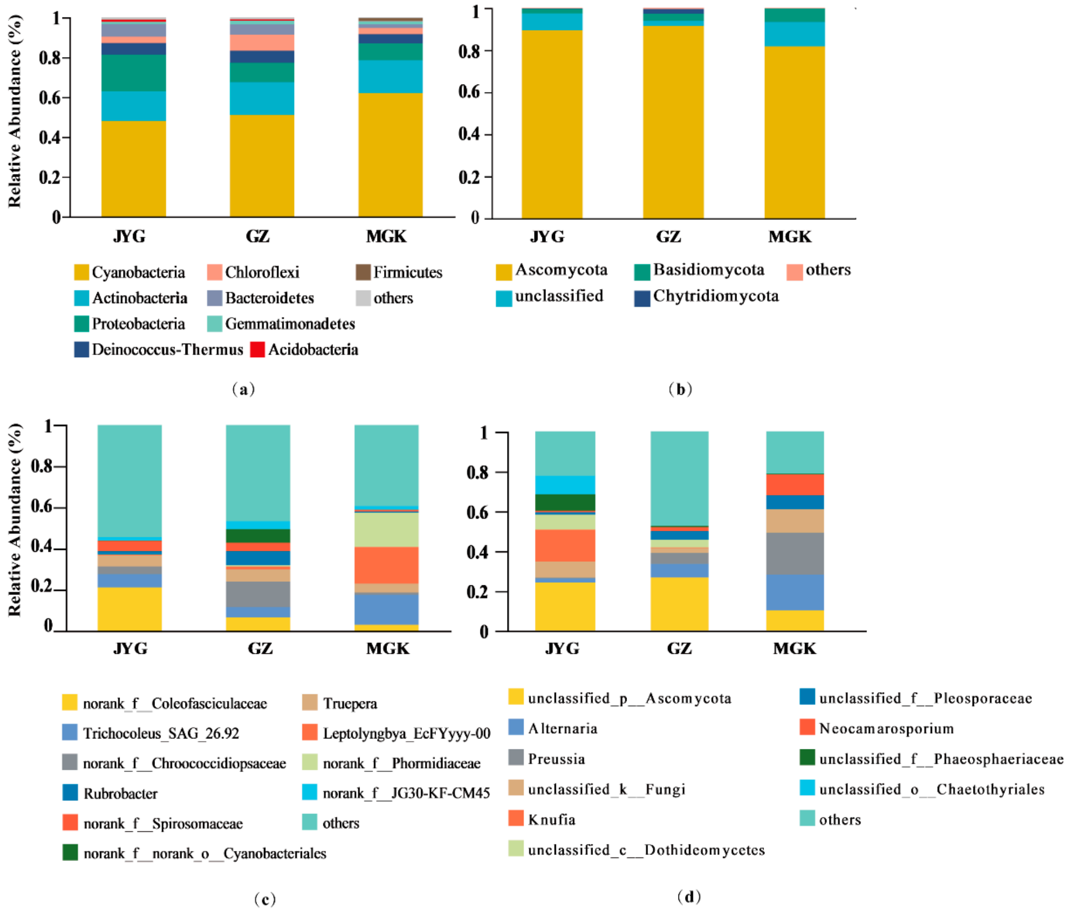

Figure S2. The relative abundance of bacterial (**a**) and fungal (**b**) phyla present in the hypolithic community. The relative abundance of bacterial (**c**) and fungal (**d**) on the genus level.

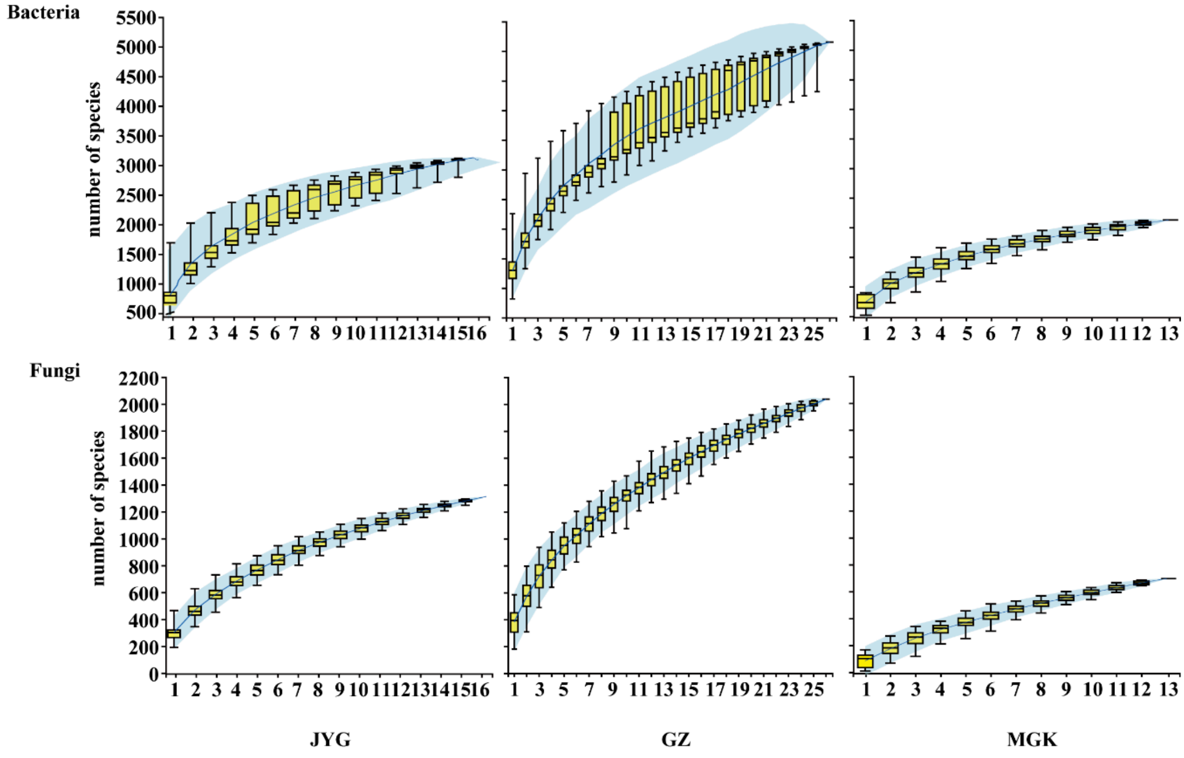

Figure S3. Cumulative Curve for bacteria and fungi at three sites. The horizontal coordinate indicates the number of samples.

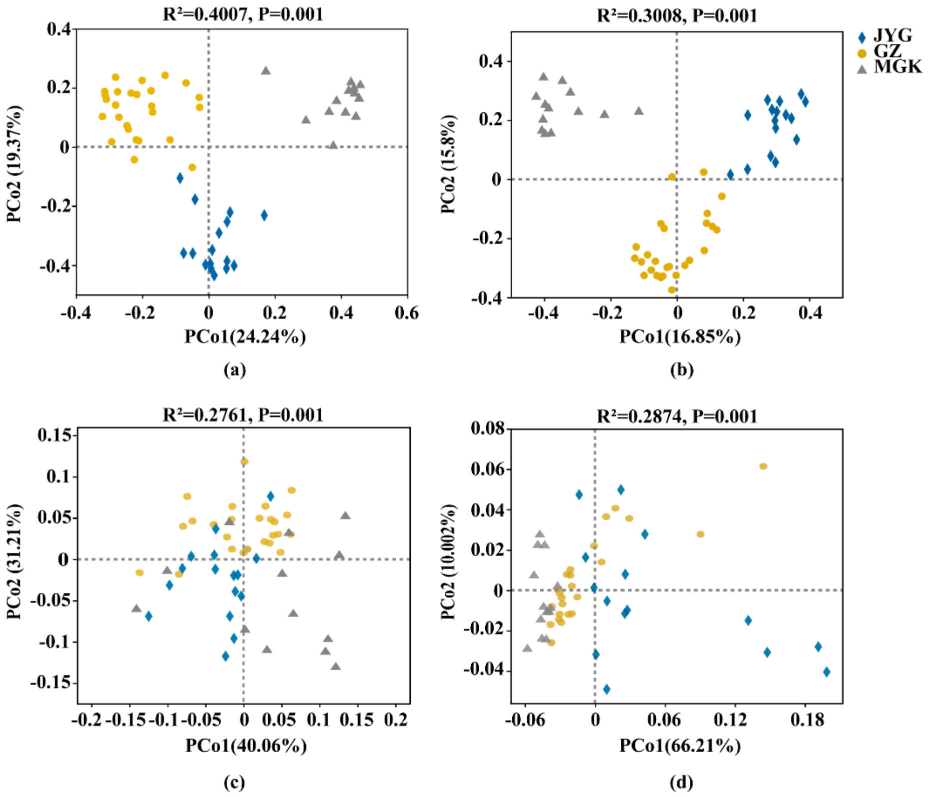

Figure S4. Principal coordinates analysis (PCoA) based on Bray-Curtis distances shows the bacterial community (**a**), fungal community (**b**), bacterial function (**c**), and fungal function (**d**) among three-site samples. The R^2 value represents the degree of explanation of sample variation by environmental factors. $p < 0.05$ indicates a significant effect.

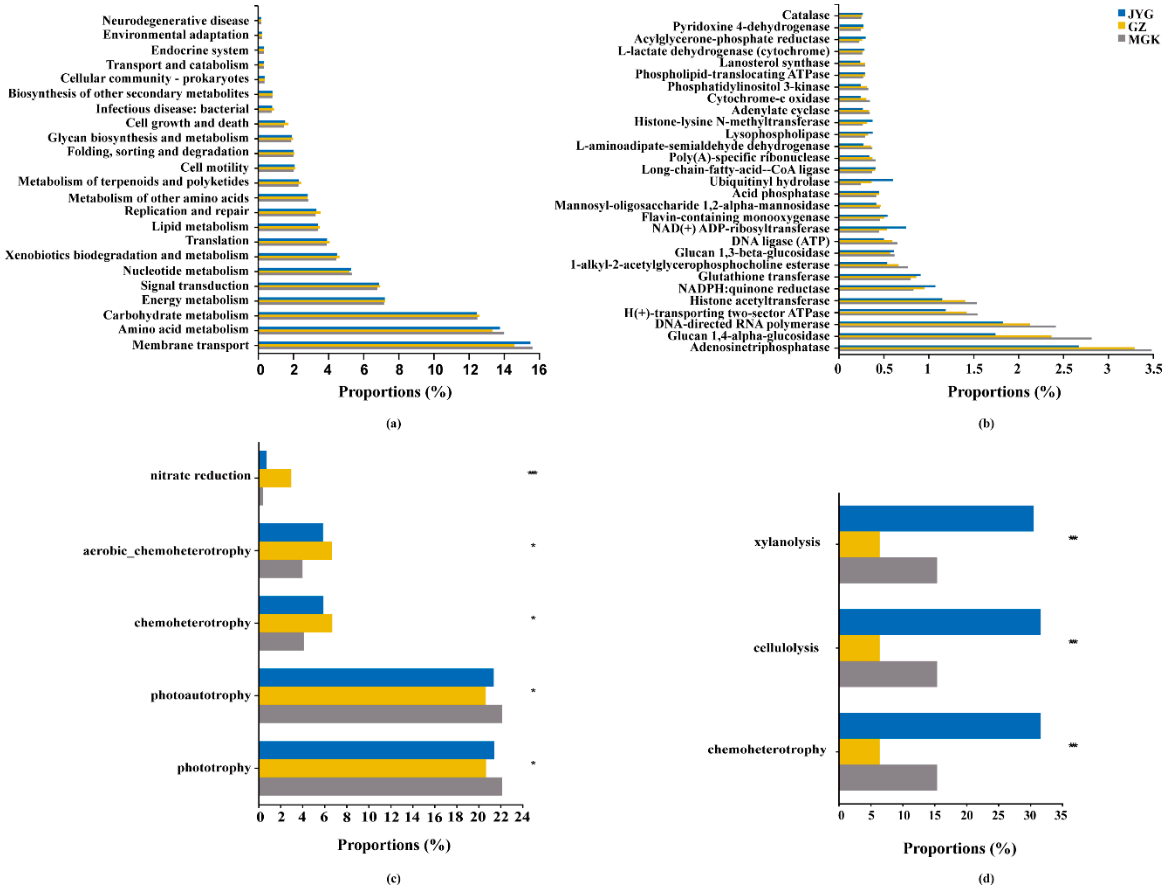

Figure S5. The analysis of the differences in the function of bacteria (**a**) and fungi (**b**) at three sites based on the PICRUSt2. The graph shows the results for P < 0.001 and the top 30 in terms of abundance. Prediction of ecologically relevant functions for bacteria (**c**) and fungi (**d**) based on FAPROTAX. * refers to $p < 0.05$; ** refers to $p < 0.01$.

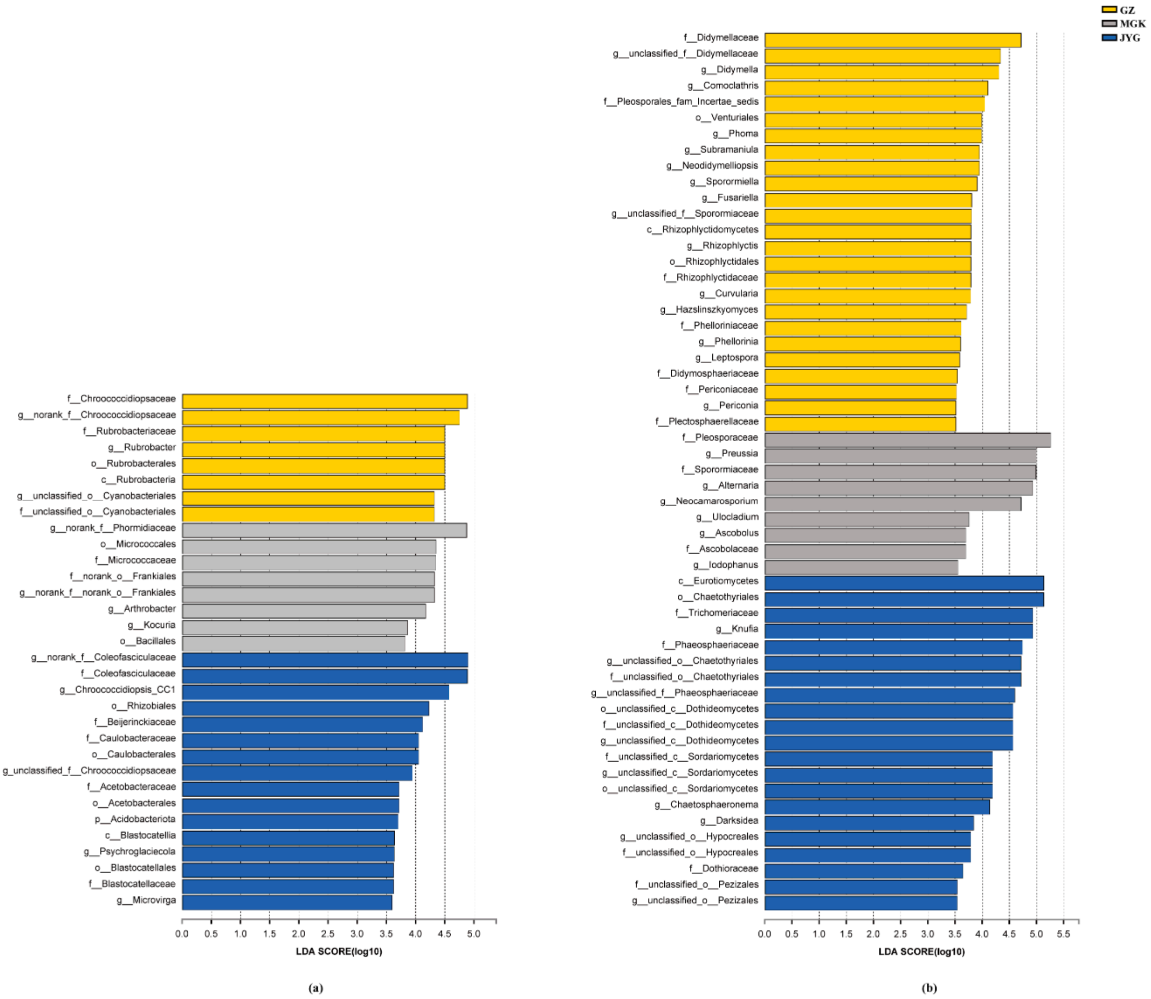

Figure S6. LDA Effect Size (LEfSe) algorithm was used on phylum to genus level to bacterial (**a**) and fungal (**b**) OTU tables. Only taxa meeting an LDA significance threshold > 3.5 are shown.
